# Occupational Attainment as a Marker of Cognitive Reserve in Multiple Sclerosis

**DOI:** 10.1371/journal.pone.0047206

**Published:** 2012-10-05

**Authors:** Omar Ghaffar, Marty Fiati, Anthony Feinstein

**Affiliations:** 1 Neuropsychiatry and Brain Sciences Programs, Department of Psychiatry, Sunnybrook Health Sciences Centre, Toronto, Ontario, Canada; 2 Neuropsychiatry Services, Ontario Shores Centre for Mental Health Sciences, Whitby, Ontario, Canada; 3 Department of Psychiatry, University of Toronto, Toronto, Ontario, Canada; Federal University of Rio de Janeiro, Brazil

## Abstract

Cognitive dysfunction affects half of MS patients. Although brain atrophy generally yields the most robust MRI correlations with cognition, significant variance in cognition between individual MS patients remains unexplained. Recently, markers of cognitive reserve such as premorbid intelligence have emerged as important predictors of neuropsychological performance in MS. In the present study, we aimed to extend the cognitive reserve construct by examining the potential contribution of occupational attainment to cognitive decline in MS patients. Brain atrophy, estimated premorbid IQ, and occupational attainment were assessed in 72 MS patients. The Minimal Assessment of Cognitive Functioning in MS was used to evaluate indices of information processing speed, memory, and executive function. Results showed that occupational attainment was a significant predictor of information processing speed, memory, and executive function in hierarchical linear regressions after accounting for brain atrophy and premorbid IQ. These data suggest that MS patients with low occupational attainment fare worse cognitively than those with high occupational attainment after controlling for brain atrophy and premorbid IQ. Occupation, like premorbid IQ, therefore may make an independent contribution to cognitive outcome in MS. Information regarding an individual's occupation is easily acquired and may serve as a useful proxy for cognitive reserve in clinical settings.

## Introduction

Forty to sixty percent of multiple sclerosis (MS) patients are affected by cognitive impairment of sufficient severity to impede their quality of life [1]. Despite this considerable morbidity, why some patients develop cognitive dysfunction and others do not remain incompletely understood.

Cognitive reserve is defined as the difference between observed neuropsychological performance and performance predicted on the basis of brain pathology [2]–[4]. Life experiences that seem to delay or limit cognitive dysfunction have been delineated in Alzheimer disease [5]. More recently, the concept of cognitive reserve has been applied to MS (reviewed in [1]). Premorbid intelligence [6]–[10] and cognitive leisure [11] were shown to independently account for variance in cognitive function that was unexplained by MS. These data suggest that the dissociation of brain pathology from cognitive function in MS may be resolved by a more complete understanding of the elements that comprise cognitive reserve. In the present study, we aimed to extend the cognitive reserve construct by examining the potential contribution of occupational attainment to cognitive decline in MS.

## Materials and Methods

### Recruitment

MS patients were recruited consecutively from two university MS clinics. No financial compensation was offered. Inclusion criteria consisted of a diagnosis of MS by the modified Macdonald criteria [12], fluency in English, and the ability to provide informed consent. Exclusion criteria consisted of age >65 years, corticosteroid treatment in the 4 weeks preceding assessment, history of any additional neurological diagnosis or medical illness that could influence cognition including head injury with loss of consciousness of any duration. Demographic and disease variables including the Expanded Disability Severity Scale (EDSS, [13]) and Multiple Sclerosis Functional Composite Score [14] were collected on the same day as cognitive reserve variables, neuropsychological testing, and magnetic resonance imaging described below.

### Cognitive reserve variables

#### Premorbid IQ

The American National Adult Reading Test (AMNART) was used to estimate premorbid verbal IQ (VIQ) corrected for educational attainment [15]. Tests of acquired vocabulary knowledge such as the AMNART have been used extensively in the cognitive reserve literature (e.g. [16]) because they provide estimates of intellectual enrichment independent of cerebral pathology [17].

#### Occupational attainment

The occupation held for the longest period was determined during patient interview with information supplemented from medical records. Occupations were classified according to U.S. Department of Labors' Dictionary of Occupational Titles (DOT), Revised 4^th^ edition [18] and divided into *high* or *low* attainment based on occupational category [19]. *High* occupational attainment was defined as professional, technical, and managerial occupations (DOT code 0-1). *Low* occupational attainment was defined as clerical/sales, agricultural/fishery/forestry, processing, machine trades, benchwork, and structural occupations (DOT code 2–8). Similar divisions of occupation have been utilized the cognitive reserve literature ([19], [20], reviewed in [21]).

### Neuropsychological assessment

Three cognitive domains from the Minimal Assessment of Cognitive Function in MS (MACFIMS) battery [22] were assessed: 1. Information processing speed was evaluated with the Paced Auditory Serial Addition Task (PASAT, 3 s version) and the Symbol Digit Modalities Test (SDMT); 2. Memory was assessed with the California Verbal Learning Test – II (CVLT-II); and 3. Executive function was tested with the Delis-Kaplan Executive Function Systems Sorting Test (DKEFS). These indices were selected on the basis of previous research on cognitive reserve in MS [6]–[11] and Alzheimer's disease [23]. Raw scores for each index were converted to age- and education-corrected z-scores based on the recommended [22] published normative values. Mean z-scores were calculated for the CVLT-II (mean of *total learning* and *delayed recall* norm-referenced z-scores) and DKEFS (mean of *number of correct sorts* and *description score* norm-referenced z-scores).

### Brain volume

Brain parenchymal fraction (BPF) was used as a marker of brain pathology [24]. Images were acquired on a 3T Signa scanner (GE Medical Systems, Milwaukee, WI) with a quadrature head coil [25]. Sequences consisted of: 1. A high resolution T1-weighted acquisition (axial 3D inversion recovery (IR)-prepped fast spoiled gradient echo (FSGR) sequence with a 3.2 ms echo time (TE), 7 ms repetition time (TR), 300 ms inversion time (TI), 15° flip angle, 22 cm×16.5 cm field of view (FOV), 256×192 matrix, 1.4 mm slice thickness, 2 excitations (NEX)); 2. A PD/T2-weighted sequence (2D interleaved axial dual-echo fast-spin echo with a TE of 20 ms and 102 ms, TR of 2900 ms, echo train length of 12, 22 cm×16.5 cm FOV, 256×192 matrix, 3.0 mm slice thickness, 2 NEX); and 3.A fluid-attenuated inversion recovery (FLAIR) sequence (2D T2FLAIR with a TE of 140 ms, a TR of 9300 ms, a TI of 2200 ms, 256×192 matrix, 22×16.5 cm FOV, 3.0 mm slice thickness, 2 NEX).

FSL's FLIRT [26] was used to coregister and reslice the PD/T2 and FLAIR in T1 space. Brain extraction was completed using BET [27] on PD/T2 and FLAIR images. Details of the protocol for tissue class segmentation [28] and white matter lesion segmentation are described previously [25], [29]. Volumes of grey matter, white matter, and CSF were obtained from segmented T1-weighted images after subtracting lesions and correcting for head size [30]. Whole brain volume was measured by BPF, where BPF  =  [(gray matter volume)+(white matter volume)]/[(gray matter volume)+(white matter volume)+(CSF volume)].

### Statistics

Statistical analyses were performed using IBM-SPSS 20.0 with alpha set at 0.05. Relationships between patient characteristics and cognitive reserve variables were evaluated with independent t-tests or Mann-Whitney tests and Pearson or Spearman's correlations. Correlations of BPF and cognitive outcomes in *low* and *high* occupational groups were compared with Fisher transformation after statistically-adjusting for VIQ. Hierarchical regression was used to assess the contribution of occupation (step 3) to cognitive outcomes after accounting for brain volume (step 1) and VIQ (step 2).

### Standard protocol approvals, registrations, and patient consent

This study was approved by the Research Ethics Boards of St. Michael's Hospital and Sunnybrook Health Sciences Centre, University of Toronto, in accordance with the recommendations for biomedical research in humans in the 1964 Declaration of Helsinki. All participants provided informed, written consent.

## Results

Table 1 shows demographic and disease variables for the 72 MS patients. Overall, 26 patients (36.1%) were cognitively-impaired by the MACFIMS criteria [22]. Forty MS patients (55.6%) were classified with *high* occupational attainment and 32 (44.4%) with *low*. The two groups did not differ with respect to employment status. Those in the *high* occupation group had greater VIQ and more years of education than the *low* group (table 1). A greater proportion of individuals in the high occupation group were receiving disease-modifying treatment at the time of testing. Among demographic and disease variables, VIQ was associated only with years of education (ρ = 0.50, p<0.001 ). The frequency of overall cognitive impairment did not differ between the *high* (30.0%) and *low* (43.8%) occupation groups (*X* ^2^ = 1.457, p = 0.324).

**Table 1:** Demographic and disease variables.

		Occupational Attainment			
	Sample	Low	High	Statistic	p
	n = 72	n = 32	n = 40		
Age, y^a^	43.0±9.7	41.1±8.5	44.6±10.5	t,df = 1.534,70	0.130
Female, n (%) b	46 (63.9)	21 (65.6)	25 (62.5)	*χ^2^* = 0.075	0.784
Education, y c	16.0 (13.0–17.0)	13.0 (12.0–15.0)	16.0 (16.0–18.0)	U = 1116.0	<0.01*
Disease course, n (%)^b^				?^2^ = 2.189	0.335
RR	59 (81.9)	24 (75.0)	35 (87.5)		
SP	9 (12.5)	6 (18.8)	3 (7.5)		
PP	4 (5.6)	2 (6.3)	2 (5.0)		
Disease duration, y c	6.5 (2.8–10.1)	6.0 (2.5–9.0)	7.0 (3.0–11.0)	U = 690.0	0.312
Age at diagnosis, y a	35.0±9.3	33.8±8.8	36.6±9.6	t,df = −1.218,67	0.227
DMD n (%) b	39 (54.2)	12 (37.5)	27 (67.5)	?^2^ = 6.445	0.011*
EDSS c	4.0 (3.0–6.0)	4.0 (3.1-6.0)	3.5 (2.5–6.0)	U = 526.5	0.195
9HPT c	21.0 (18.0–25.0)	23.0 (18.2–25.5)	20.0 (17.9–24.3)	U = 505.5	0.171
TWT c	4.4 (4.0–6.9)	4.8 (4.1–7.6)	4.1 (3.4–6.3)	U = 420.0	0.132
BDI-II c	12.0 (5.0–23.0)	14.5 (6.8–27.8)	12.0 (3.5–19.5)	U = 479.5	0.340
VIQ c	116.4 (112.2–119.4)	112.7 (111.4–118.3)	117.8 (114.4–120.3)	U = 422.0	0.013*
Employed n (%) b	51 (70.8)	20 (62.5)	31 (77.5)	?^2^ = 1.936	0.164

a T-test (values are means ± SD).

b χ^2^ test.

c Mann-Whitney test (values are median [IQR]).

*Abbreviations:* RR – relapsing-remitting; SP – secondary progressive; PP – primary progressive; DMD – Disease-modifying medication; EDSS – Expanded Disability Severity Scale; 9HPT – 9-hole pegboard test; TWT – timed walking test; BDI-II – Beck Depression Inventory-II; VIQ – verbal IQ.

**Figure 1:**
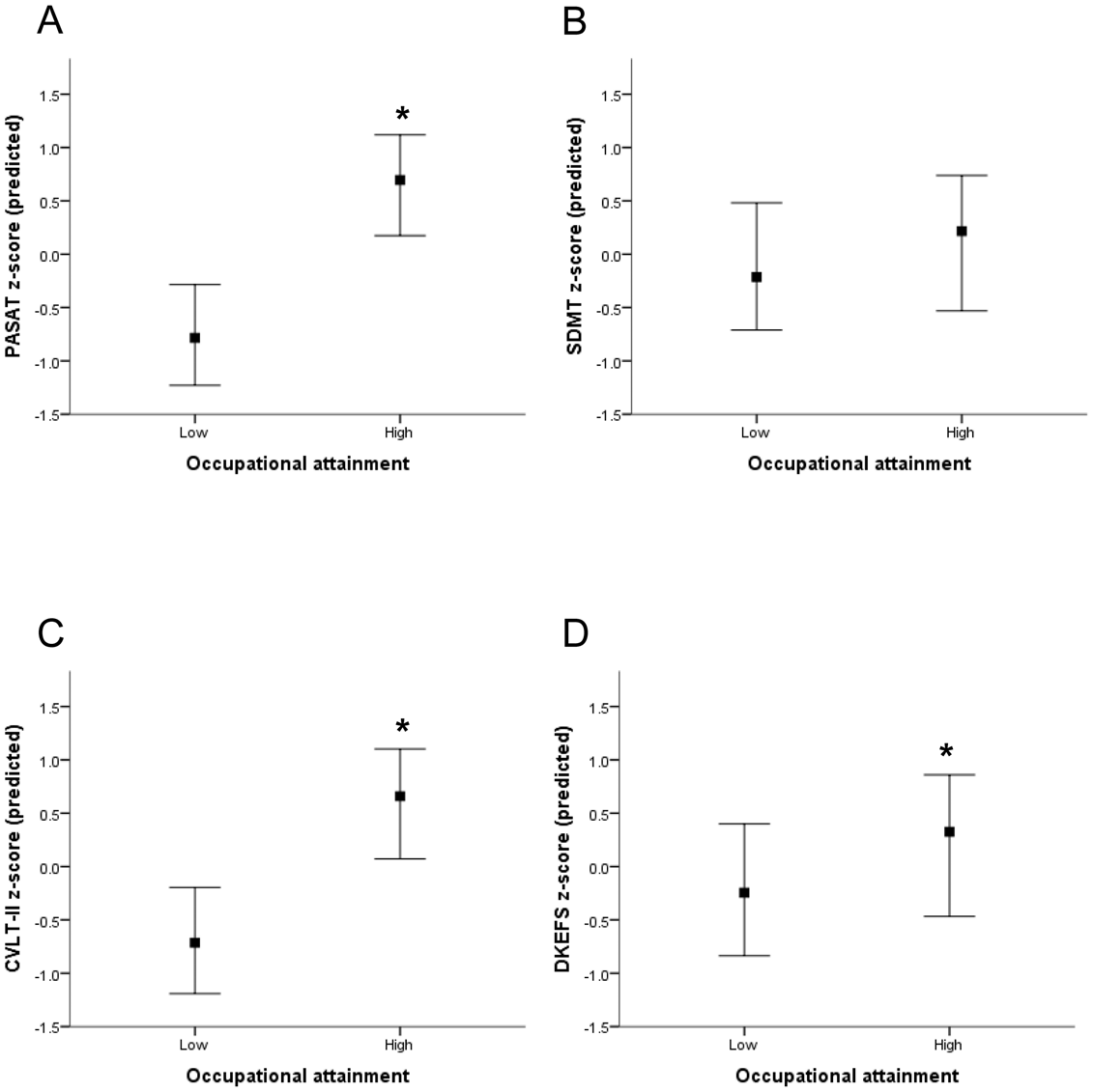
Occupational attainment and cognitive outcome in MS patients. Median and interquartile range for standardized predicted scores on the PASAT (A), SDMT (B), CVLT-II (C), and DKEFS (D) are shown in patients with *low* and *high* occupational attainment. Occupational attainment was a significant predictor of the PASAT, CVLT-II, and DKEFS after accounting for BPF and VIQ by hierarchical regression *p<0.05.

Correlations of neuropathology (BPF) with cognitive outcome differed between *high* and *low* occupational groups after adjusting for VIQ (PASAT, z = 3.26, p = 0.001; SDMT, z = 3.71, p = 0.001; CVLT-II, z = 3.31, p = 0.001; DKEFS, z = 3.31, p = 0.001). The contribution of occupation to cognitive outcome was assessed by hierarchical regression. BPF (step 1), VIQ (step 2), and occupation (step 3) were entered as predictors of performance on the PASAT, SDMT, CVLT-II, and DKEFS. The full regressions accounted for the following variance: 20.4% for the PASAT, 17.7% for the SDMT, 33.2% for CVLT-II, and 19.2% for the DKEFS. BPF and VIQ predicted performance on all four cognitive tests in the full regressions (Table S1). Occupation accounted for additional variance in the PASAT, CVLT-II, and DKEFS, but not the SDMT (table 2 and figure 1). Years of education did not predict any cognitive outcome in the regressions (step 1 =  BPF, step 2 =  VIQ, step 3 =  years of education, step 4 =  occupation), but the statistical significance of occupation was lost for the DKEFS when education was included. Disease-modifying treatment did not predict any cognitive outcome in the regressions (step 1 =  BPF, step 2 =  VIQ, step 3 =  disease-modifying treatment, step 4 =  occupation), and the statistical significance of occupation was unchanged with the inclusion of this variable.

**Table 2:** Association of occupational attainment and cognitive outcomes in MS by hierarchical regression.

	Occupational attainment (step 3)^a^				Overall Model		
Dependent variable	t	Partial *r*	Δ*R^2^*	*p*	*F*	*R^2^*	*p*
PASAT	2.176	0.255	0.055	0.033*	5.808	0.204	0.001*
SDMT	0.563	0.068	0.004	0.575	4.856	0.177	0.004*
CVLT-II	2.632	0.304	0.068	0.011*	11.246	0.332	<0.001*
DKEFS	2.002	0.236	0.048	0.049*	5.366	0.192	0.002*

a Step 1 was BPF and step 2 was VIQ. Additional information is provided in Table S1.

* p<0.05.

## Discussion

This study is the first to our knowledge to report an association between occupational attainment and cognitive outcome in MS. After accounting for brain atrophy and premorbid IQ, occupational attainment added significant variance to indices of information processing speed (PASAT), memory (CVLT-II), and executive function (DKEFS). These data suggest that MS patients with low occupational attainment fare worse cognitively than those with high occupational attainment after controlling for brain atrophy and premorbid IQ. The implication is that occupational attainment, like premorbid IQ [6], [7], [8], [9] and cognitive leisure [11], makes an independent contribution to cognitive reserve in MS.

The concept of cognitive reserve emerged from epidemiological research in dementia [3], [5], [31]. The cognitive reserve theory helped explain the weak affiliation of neuropathology with cognition, potentially resolving conundra such as normal ante-mortem neuropsychological function in elders who met full pathological criteria for Alzheimer disease at autopsy [32]. Over the last two decades, the notion that life experiences can modify age-related neuropathological changes has garnered considerable empirical support. Because cognitive reserve cannot be directly quantified, proxy measures have served as surrogates. Pre-morbid IQ, education, occupational attainment, and cognitively-stimulating leisure activities are considered markers of cognitive reserve, for example, because they can account for inter-individual differences in the incidence of dementia when other relevant variables are controlled [3], [5], [31]. A meta-analysis of over 29 000 participants found that high cognitive reserve was associated with a 50% decrease in the risk of incident dementia [21]. The benefits of high cognitive reserve were dose-dependent and comparable in magnitude to risk reduction with blood pressure control and anti-inflammatory medication. The association between enriching life experiences and cognitive preservation in the face of neuropathology has been extended to stroke [33] and traumatic brain injury [34].

A handful of studies have examined cognitive reserve in MS. In the first, Sumowski et al. [9] compared the performance of 58 MS patients to healthy controls on the SDMT, the PASAT, and indices of verbal memory derived from the Logical Memory subtests of the Weschler Memory Scale-Revised. MS patients with high cognitive reserve, estimated with a word-reading proxy of VIQ, scored similarly to healthy controls on the PASAT and verbal memory tests. Low cognitive reserve, in contrast, was associated with significantly poorer measures on these cognitive outcomes in the MS patients. Of note is that the protective effect of high reserve did not apply to the SDMT–MS patients, irrespective of high or low cognitive reserve, performed significantly worse than controls on this task. In our study, occupational attainment was also unassociated with the SDMT yet was an important predictor of the PASAT. Both measures are considered indices of information processing speed. Dissociation between the SDMT and PASAT with respect to cognitive reserve has been suggested to relate to the former mobilizing a more limited range of cognitive strategies [9]. The PASAT, in contrast, tests not only processing speed and working memory but also requires numeric calculation [17]. It is amenable to different cognitive strategies such as “chunking” of stimulus dyads [35]. The relative independence of the SDMT from proxies of cognitive reserve could also explain why it is generally the most robust correlate of brain pathology in MS [1].

More recent research has partitioned the extent of cognitive change that can be attributed to measurable brain pathology versus cognitive reserve. Sumowski et al. [6], [7] incorporated a brain atrophy measure, namely, third ventricular width, in hierarchical regressions predicting cognitive outcome in MS. With the inclusion of atrophy, premorbid IQ remained a significant determinant of information processing speed (quantified by the average of the SDMT and PASAT) and verbal memory (measured with the Selective Reminding Test). Higher reserve thus lessened the effect of brain atrophy on cognition in MS patients.

Moving beyond IQ as a proxy of cognitive reserve in MS, premorbid cognitive leisure was surveyed in 36 patients by the same research group using a questionnaire that retrospectively probed activities such as reading, hobbies, playing a musical instrument, producing art, etc. in patients' early 20 s [11]. Participation in these intellectually-enriching activities was found to be positively correlated with cognitive outcome (a composite measure of verbal memory and information processing speed) after controlling for brain atrophy, premorbid IQ, and education.

Our data confirm previous studies linking premorbid IQ to cognitive function in MS. We found significant associations of VIQ with information processing speed (PASAT) and memory (CVLT-II) when controlling for BPF. We used BPF as our measure of brain atrophy rather than third ventricular width given that it furnishes a direct index of whole brain volume corrected for lesion volume. Of note is that we also extended the range of cognitive indices linked to cognitive reserve by including a measure of executive function, the DKEFS, an important determinant of individuals' daily functioning, including medical and financial decision-making [36]. By doing so, we provide a more comprehensive measure of what cognitive reserve entails in MS. The executive function finding is consistent with what has been reported in cognitive reserve studies of Alzheimer disease [23].

The role of education in cognitive reserve remains unclear. Evidence in the aging and dementia literature suggest that premorbid IQ may furnish a more robust measure of reserve than education [37], [38]. A MS study that lacked brain atrophy measurement reported that education accounted for 2% of the variance in cognitive outcome independent of premorbid intelligence [8]. The inclusion of education as a putative reserve variable in other MS studies has been inconsistent. In our study, education was not a predictor of cognitive outcome. Methodological issues cannot be excluded from this finding, however. In particular, standardizing cognitive variables to normative values correcting for age and education may have a diminished an education effect. Also, quality of schooling, which we did not measure, may contribute to cognitive reserve [39] in addition to years of education. Finally, in our sample of relatively well-educated individuals, a ceiling effect could have obscured a significant effect of education. It is notable that the independent contribution of occupational attainment to executive function lost statistical significance with the inclusion of education in the regression model. Possibly, the diverse life experiences that influence cognitive reserve individually impact different neuropsychological functions to varying degrees. Tentative support for this notion may be found in the present study and in earlier work [9] reporting associations of cognitive reserve markers with performance on the PASAT but not on the SDMT.

Our study is not without its limitations and requires replication. While our data and earlier studies [20], [40] support an effect of occupational attainment on cognitive reserve that is separate from that imparted by innate intelligence and education, the causal mechanisms and multi-directional pathways that may exist between these variables remain undefined. The occupational groups were well matched on age of onset, disease duration, disease course, and physical disability. Nonetheless, the possibility that individuals with worse disease early on were forced into less cognitively-demanding jobs cannot be ruled out without longitudinal data that has thus far been lacking in the MS-cognitive reserve literature. Fatigue, a common MS symptom, can also influence cognitive outcomes but was not measured in this study. Likewise, data regarding leisure activities was not collected. Another potential limitation is that the groups were not matched with respect to disease-modifying treatment at the time of cognitive assessment. Evidence for an effect of these medications on cognition is generally weak, however [reviewed in 1], and we did not detect an effect on cognitive outcomes. Furthermore, because educational and occupational attainment comprise part of what defines socioeconomic status, the potentially confounding effects of income, access to healthcare, etc. are difficult to disentangle. This challenge is not unique to our study [4].

In summary, our data emphasize the importance of considering cognitive reserve in determining MS patients' cognitive status. In doing so, we add occupational attainment to the small list of variables that define what cognitive reserve engenders. Unlike imaging variables such as BPF or third ventricular width, information regarding an individual's occupation is easily acquired in clinical settings. Whether this information can assist clinicians in predicting future cognitive impairment in MS patients awaits further study.

## Supporting Information

Table S1 **BPF, VIQ, and occupational attainment as predictors of cognitive outcome in MS.**
(XLS)
